# From the Guiana Highlands to the Brazilian Atlantic Rain Forest: four new species of *Selaginella* (Selaginellaceae – Lycopodiophyta: *S*.* agioneuma*, *S. magnafornensis*, *S*. *ventricosa*, and *S. zartmanii*)

**DOI:** 10.7717/peerj.4708

**Published:** 2018-05-11

**Authors:** Iván A. Valdespino, Christian A. López, Adriel M. Sierra, Jorge Ceballos

**Affiliations:** 1 Departamento de Botánica, Facultad de Ciencias Naturales, Exactas y Tecnología; Sistema Nacional de Investigación (SNI), Universidad de Panamá, Panamá, Panama; 2 Departamento de Botánica, Facultad de Ciencias Naturales, Exactas y Tecnología, Universidad de Panamá, Panamá, Panama; 3 Departamento de Biodiversidade, Instituto Nacional de Pesquisas da Amazônia, Manaus, Brazil; 4 Smithsonian Tropical Research Institute, Panamá, Panama

**Keywords:** Amazon, Atlantic forest, Idioblasts, Laminar flap, Lycophytes, Papillae, Prickles, Stomata, Tooth-like

## Abstract

We describe four new species in the genus *Selaginella* (i.e., *S*.* agioneuma, S. magnafornensis*, *S*. *ventricosa*, and *S*. *zartmanii*) from Brazil, all presently classified in subg. *Stachygynandrum*. For each of the new taxa we discuss taxonomic affinities and provide information on habitat, distribution, and conservation status. In addition, line drawings and scanning electron microscope (SEM) images of stems sections, leaves, and spores (when present) are included. *Selaginella agioneuma* and *S. magnafornensis* are from the State of Espíritu Santo where they inhabit premontane to montane Atlantic rain forests in the Reserva Biológica Augusto Ruschi and Parque Estadual Forno Grande, respectively. *Selaginella ventricosa* was collected in upper montane forests at Parque Nacional Serra da Mocidade, State of Roraima and *S. zartmanii* in premontane Amazon rain forests on upper Rio Negro at Mpio. São Gabriel da Cachoeira, Amazonas State in both Serra Curicuriari and the Morro dos Seis Lagos Biological Reserve.

## Introduction

*Selaginella* P. Beauv. (1804: 478, Selaginellaceae – Lycopodiophyta) is the most species-rich genus of extant lycophytes (Valdespino, 2015a; PPG I, 2016; Valdespino, 2016; Valdespino, 2017a) and one of the oldest seedless vascular plants with fossils dating approximately 333–350 mya (Banks, 2009) and 330–390 mya (Hao et al., 2007; Klaus et al., 2016). The genus includes some 600–750 (PPG I, 2016; Valdespino, 2016) or ca. 800 (Zhou & Zhang, 2015) species, which Jermy (1986) and Jermy (1990) classified in five subgenera (i.e., *Selaginella*, *Tetragonostachys*, *Ericetorum*, *Heterostachys*, and *Stachygynandrum*). Recent phylogenetic analysis based on molecular data place *Selaginella* as monophyletic and sister to *Isoëtes* (Zhou et al., 2015; Weststrand & Korall, 2016a). These results have led to two contrasting infrageneric classifications. The one by Zhou & Zhang (2015) recognizes six subgenera (i.e., *Selaginella*, *Boreoselaginella*, *Pulviniella*, *Ericetorum*, *Heterostachys*, and *Stachygynandrum*), while that of Weststrand & Korall (2016a) proposes seven (i.e., *Selaginella*, *Rupestrae*, *Lepidophyllae*, *Gymnogynum*, *Exaltatae*, *Ericetorum*, and *Stachygynandrum*). Furthermore, Korall & Kenrick (2002) suggest that most extant tropical *Selaginella* species derive from recent diversifications. Arrigo et al. (2013) postulated that radiation of subg. *Tetragonostachys* sensu Jermy (1986) and Jermy (1990) in North America occurred during the early Cretaceous to late Paleocene, while Klaus et al. (2016) using Zhou & Zhang’s (2015) intrageneric classification suggested that the major divergence within the two main lineages of *Selaginella* happened in the late Permian and Early Triassic. At the same time, the modern morphology-based classification of *Selaginella* proposed by Jermy (1986) and Jermy (1990) is still widely accepted and these works in combination with Hieronymus (1901–1902) remain the foundation of taxonomic and floristic studies. Advances in *Selaginella* taxonomy have led to new discoveries resulting in a renaissance over the last sixty years (Valdespino & Zimmer, 2016). Such works have particularly improved our understanding of species distribution and diversity patterns at local, regional, and global scales. For example, Valdespino (2015a); Valdespino (2016); Valdespino (2017a); Valdespino (2017b); Valdespino (2017c) and Smith & Kessler (2018) established that the three South American countries with the richest *Selaginella* flora are in order: Venezuela (ca. 100 spp.), Brazil (89–96 spp), and Colombia (ca. 83 spp, fide Alston, Jermy & Rankin, 1981; I Valdespino, 2018, unpublished data). However, parts of these countries, especially Venezuela and Brazil remain largely unexplored botanically (e.g., the Guiana Highlands of northern South America and the Brazilian Atlantic rainforest), that most likely harbor undescribed species and surpass current species estimates for *Selaginella*.

In this paper we describe four new *Selaginella* species from Brazil based on material determined by the first author as part of his continued revisionary work of the genus for the Neotropics conducted in collaboration with the herbaria of the New York Botanical Garden and the Muséum national d’Histoire naturelle, in Paris. These novelties are named as follows: *S*. *agioneuma* Valdespino & C. López from Reserva Biológica Augusto Ruschi and *S*. *magnafornensis* Valdespino & C. López found in Parque Estadual Forno Grande, both in the State of Espíritu Santo; *S*. *ventricosa* Valdespino & C. López collected in Serra da Mocidade, State of Roraima, and *S*. *zartmanii* Valdespino, C. López & A.M. Sierra gathered from two isolated outcrops (the Serra Curicuriari and Morro dos Seis Lagos) in the extreme northwestern portion of Amazonas State. These species are all characterized by having heteromorphic vegetative leaves, non-articulate stems, and quadrangular strobili comprised of monomorphic sporophylls, which allow for their placement within subg. *Stachygynandrum* as circumscribed by Jermy (1986), Jermy (1990), and Weststrand & Korall (2016b). Consequently, of the estimated number of Brazilian *Selaginella* species, ca. 69–76 belong to subg. *Stachygynandrum*, whereas worldwide ca. 600 taxa are classified within (Weststrand & Korall, 2016b).

## Materials & Methods

This study is based on examination of herbarium specimens from INPA, NY, P, and PMA, and digitized images from P (herbarium acronyms follow Thiers, 2018). Additional virtual herbaria consulted included RB (JBRJ, 2018), Reflora (2018), and Species Link (2018). Scanning electron microscopy (SEM) micrographs from selected specimens to document leaf surfaces and sculpturing patterns of mega- and microspores (when available), from designated holotypes were prepared, viewed, digitized and post-processed according to standard techniques using an image-handling software as referenced in Valdespino (2017a) and Valdespino (2017b). Terminology, measurements, and conservation status provided for each taxon follow relevant glossaries (Punt et al., 2007; Hesse et al., 2009; Beentje, 2016), and Valdespino (2016), Valdespino (2017b), and references therein. Nonetheless, we believe it appropriate to further explain some terms such as “*idioblasts*,” which refer to those cells that differ from surrounding ones in shape (i.e., in this case they are elongate), size or function (see Beentje, 2016), regardless of the chemical composition of their intercellular or cell wall projections, which look like a continuous stripe or line. Equally, we use the term “*papilla* (pl. *papillae*)” to refer to single, discrete, round and small, nipple-like, cell surface projections. On the other hand, “*idioblast-like*” denote cells with independent papillae closely arranged in one or more rows, which may appear line-like or cells with interconnected papillae resembling a continuous stripe or line. Furthermore, we termed “*papillate cells*” those leaf cells that do not significantly differ from surrounding cells, which may have a single or many papillae and tend to be roundish to quadrangular with sinuate walls. Additionally, we define “*prickles*” or “*tooth-like*” projections as few, scabridulous, leaf surface hairs (similar to those often found in denticulate margins), which are only visible with high magnification. We abstain from referring to any of these cells or projections as being made of silica bodies because determining their chemical composition (see Prychid, Rudall & Gregory, 2004, for review) was not part of the scope of the present study. Furthermore, Valdespino (1995) discussed the term “*laminar flap*” and defined it as a laminar projection formed by a partial or continuous, incomplete fusion of the lower (adaxial) epidermis of the lower half of the dorsal sporophyll, which has the rest of that surface free and usually standing at an angle of ca. 90°. It was depicted in Valdespino (2015a, Fig. 8F) for *S*. *pellucidopuctata* Valdespino and Valdespino (2017a, Fig. 2D) for *S. hyalogramma* Valdespino.

The electronic version of this article in Portable Document Format (PDF) will represent a published work according to the International Code of Nomenclature for algae, fungi, and plants (ICN), and hence the new names contained in the electronic version are effectively published under that code from the electronic edition alone. In addition, new names contained in this work which have been issued with identifiers by IPNI will eventually be made available to the Global Names Index. The IPNI LSIDs can be resolved and the associated information viewed through any standard web browser by appending the LSID contained in this publication to the prefix “http://ipni.org/”. The online version of this work is archived and available from the following digital repositories: PeerJ, PubMed Central, and CLOCKSS.

## Taxonomic Results

**Table:** 

**Figure 1:**
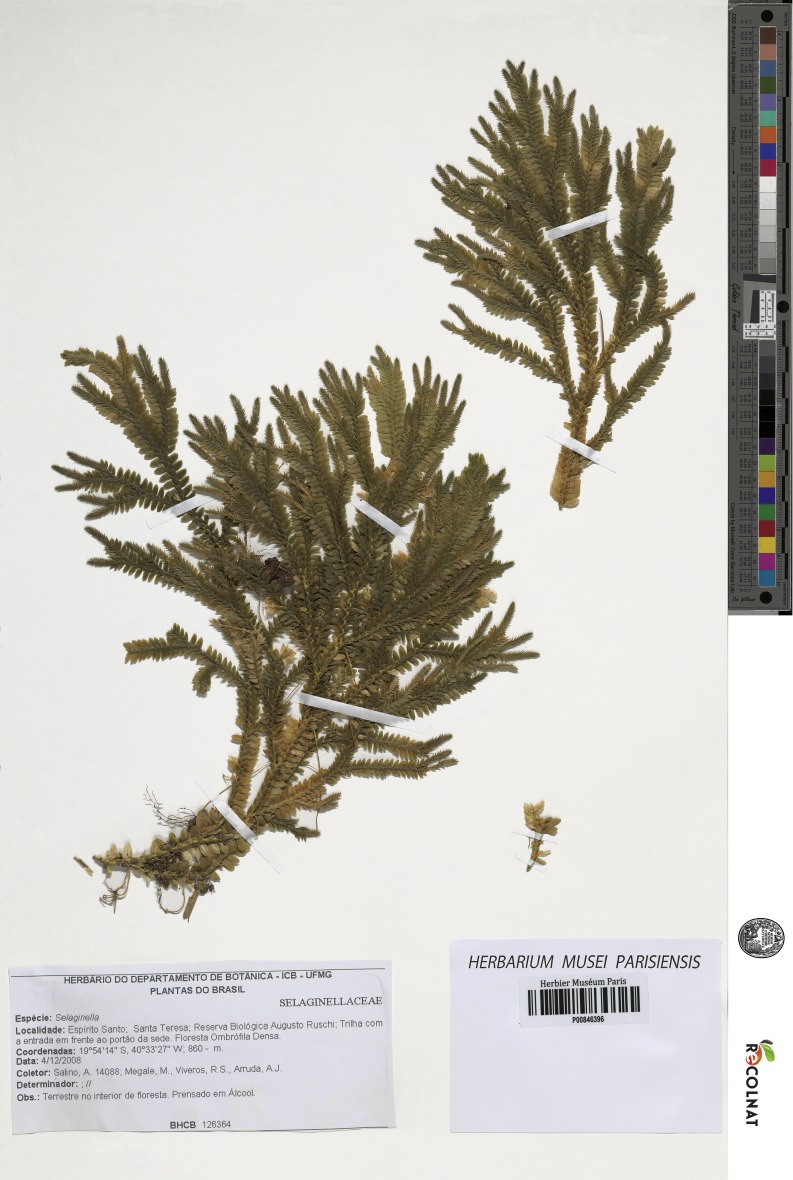
Scanned image of holotype of *S. agioneuma*. *Salino et al. 14088* (P). Image courtesy of Muséum national d’Histoire naturelle, Paris, France.

**Figure 2:**
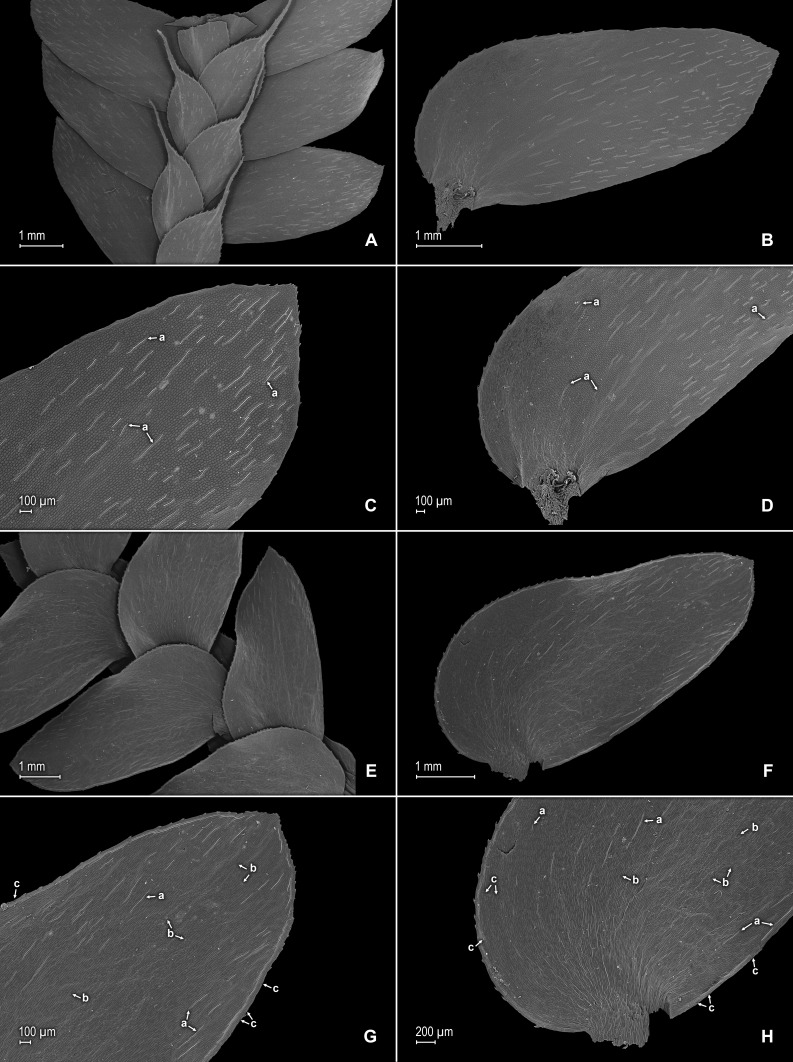
Scanning electron micrographs of branch sections and leaves of *S. agioneuma*. (A) Section of upper surface of stem branch showing lateral and median leaves. (B) Close-up of lateral leaf from stem branch, upper surface. (C) Close-up distal portion of lateral leaf (same leaf shown in B); note, elongate and papillate idioblasts (a). (D) Close-up of proximal portion of lateral leaf (same leaf shown in B); note, elongate and papillate idioblasts (a). (E) Section of lower surface of stem branch showing lateral leaves. (F) Close-up of lateral leaf from stem branch, lower surface. (G) Close-up of distal portion of lateral leaf (same leaf shown in F); note, elongate and papillate idioblasts (a) and stomata along midrib (b) and submarginal and marginal (c) portions of lamina. (H) Close-up of proximal portion of lateral leaf (same leaf shown in F); note, elongate and papillate idioblasts (a) and stomata along midrib (b) and submarginal and marginal (c) portions of lamina. (A–H) taken from holotype, *Salino et al. 14088* (P).

**Figure 3:**
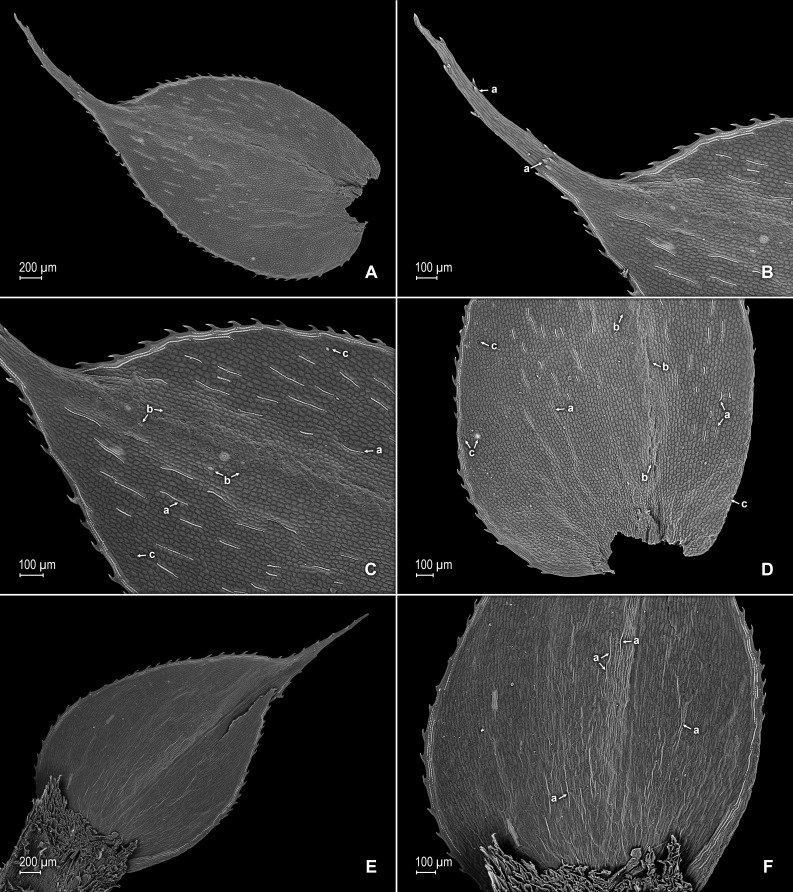
Scanning electron micrographs of median leaves of *S. agioneuma*. (A) Close-up of median leaf from stem branch, upper surfaces. (B) Close-up of distal portion of median leaf (same leaf shown in A) showing aristate apex; note tooth-like projections on arista surface (a). (C) Close-up of distal portion of median leaf (same leaf shown in A) showing section of aristate apex; note, elongate and papillate idioblasts (a) and stomata along midrib (b) and submarginal and marginal portions of lamina (c). (D) Close-up of proximal portion of median leaf (same leaf shown in A); note, elongate and papillate idioblasts (a) and stomata along midrib (b) and submarginal and marginal (c) portions of lamina. (E) Close-up of median leaf from stem branch, lower surfaces. (F) Close-up of proximal portion of median leaf (same leaf shown in E); note, elongate idioblast-like cells on leaf lamina (a). (A–F) taken from holotype, *Salino et al. 14088* (P).

**Figure 4:**
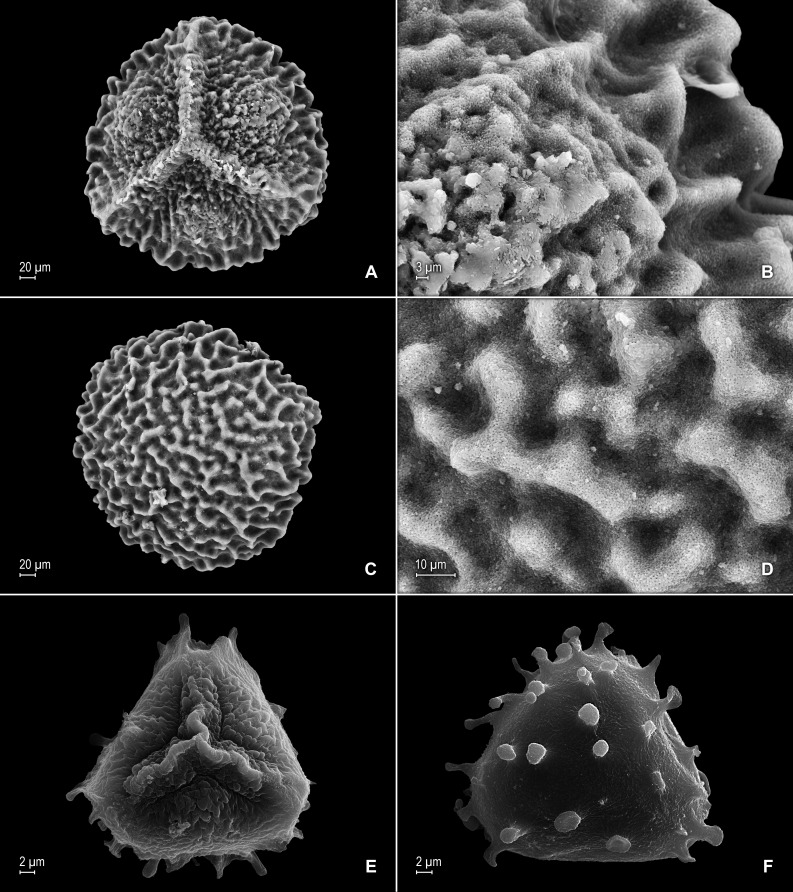
Scanning electron micrographs of mega- and microspores of *S. agioneuma*. (A) Megaspore, proximal face. (B) Close-up of megaspore, proximal face. (C) Megaspore, distal face. (D) Close-up of megaspore, distal face. (E) Microspore, proximal face. (F) Microspore, distal face. (A–F) taken from holotype, *Salino et al. 14088* (P).

***Selaginella agioneuma*** Valdespino & C. López, sp. nov.
(Figs. 1–4)

**Type:–**BRAZIL. Espíritu Santo: Santa Teresa, Reserva Biológica Augusto Ruschi, trilha com entrada em frente ao portão da sede, 19°54′14″S, 40°33′27″W, 860 m, 4 Dec 2008, *A. Salino, M. Megale, R.S. Viveros & A.J. Arruda 14088* (holotype: P!; isotypes: BHCB-n.v., fragment PMA!).

**LSID:**77178084-1.

**Diagnosis:–***Selaginella agioneuma* differs from *S*. *contigua* Baker by its dentate (vs. ciliate) median leaves with entire outer bases (vs. tufted with two to five cilia) and aristate apices with each arista 0.5–1.5 mm (vs. apices acuminate to aristate with each acumen or arista 0.05–0.7 mm) long, oblong or broadly ovate-oblong (vs. narrowly oblong) lateral leaves, each 4.0–7.5 × 1.5–3.5 mm (vs. 7.0–10 × 2.0–2.7 mm) with dentate (vs. ciliate at least along proximal 1∕2) acroscopic bases, broad and abruptly tapering (vs. narrow and gradually tapering) apices ending in an acute tip, and axillary leaf margins denticulate or the inner margins denticulate along proximal 1∕2 and entire distally and the outer margins denticulate throughout (vs. both margins ciliate at least along proximal 1∕3–1∕2, otherwise denticulate to entire).

**Description:–***Plants* terrestrial. *Stems* decumbent to ascending, stramineous, 15–30 cm long, 1.0–1.5 mm diam., non-articulate, not flagelliform or stoloniferous, 2- to 3-branched. *Rhizophores* ventral and dorsal, borne on proximal 1∕3–1∕2 of stems, stout, 0.4–0.8 mm diam. *Leaves* heteromorphic throughout, chartaceous to coriaceous, strongly imbricate, upper surfaces smooth and green, lower surfaces smooth and green and yellowish green. *Lateral leaves* perpendicular to the stems or slightly ascending, oblong or broadly ovate-oblong, 4.0–7.5 × 1.5–3.5 mm; bases rounded to semicordate, glabrous, acroscopic bases strongly overlapping stems, basiscopic bases free from stems; acroscopic margins hyaline in a band two to four cells wide with the cells elongate, straight-walled and papillate parallel to margins, papillae in one row over each cell lumen, denticulate along proximal 1∕2, then becoming entire and again denticulate along distal 1∕4 or denticulate throughout, basiscopic margins narrowly hyaline, comprised of quadrangular, sinuate-walled, glabrous cells, entire along proximal 3∕4, otherwise denticulate on distal 1∕4; apices acute to apiculate, variously tipped by three to five teeth; upper surfaces comprised of quadrangular or rounded, sinuate-walled cells, with many idioblasts on both sides of the midribs and without stomata or sometimes with submarginal to marginal stomata along basiscopic margins, lower surfaces comprised of elongate, sinuate-walled cells, papillate idioblast on both side of the midribs, papillae in one row over each cell lumen, with stomata in six to nine irregular rows along midribs and sparsely distributed on submarginal to marginal portion of the acroscopic half of the leaf. *Median leaves* ascending, ovate to broadly ovate, 2.3–3.2 × 1.5–2.2 mm; bases subcordate, glabrous; margins hyaline in a band two to four cells wide, the cells elongate, straight-walled and papillate parallel to margins, papillae in 1 row over each cell lumen, inner margins dentate to short-ciliate throughout, the outer margins entire along proximal 1∕2, dentate to short-ciliate along distal 1∕2; apices long-aristate, each arista 1∕4–1∕2 the length of the laminae (0.5–1.5 mm long), dentate and variously tipped by one to three teeth and with one to four short tooth-like projections on its upper surfaces; upper surfaces with conspicuous idioblasts, comprised of quadrangular, rectangular or rounded, sinuate-walled cells, with stomata in two to four rows along the midribs and sparsely present on submarginal portion along outer and inner halves of the laminae, lower surfaces comprised of elongate, sinuate-walled cells, without stomata. *Axillary leaves* similar to lateral leaves or more ovate or narrowly ovate, 4.0–5.0 × 1.5–3.0 mm; bases subcordate, glabrous; margins hyaline, denticulate or the inner margins denticulate along proximal 1∕2 and entire distally and the outer margins denticulate throughout; apices acute to apiculate, variously tipped by one to three teeth; both surfaces as in lateral leaves. *Strobili* terminal on main stem and branch tips, quadrangular, 1.0–2.0 cm. *Sporophylls* monomorphic, without a laminar flap, each with a well developed and glabrous keel (as observed with stereomicroscope, SM) along midribs, ovate-lanceolate, broadly ovate or ovate-deltate, 1.2–2.6 × 0.8–1.5 mm; bases rounded; margins broadly hyaline, dentate; apices acuminate to aristate, each acumen or aristae 0.2–0.5 mm long, tipped by one or two teeth; *dorsal sporophylls* with upper surfaces green and cells as in median leaves, including idioblasts, lower surfaces silvery green and comprised of elongate, sinuate-walled cells; *ventral sporophylls* with both surfaces hyaline, comprised of elongate, sinuate-walled cells and idioblasts. *Megasporangia* in two ventral rows or with few also intermixed on distal portion of dorsal rows; *megaspores* lemon yellow, rugulate on proximal faces with verrucate and perforate microstructure, closely reticulate on distal faces with verrucate and perforate microstructure (Figs. 4A–4D), 280–295 µm. *Microsporangia* in two dorsal rows or often intermixed distally on dorsal rows with megasporangia; *microspores* orange, rugulate on proximal faces with slightly verrucate microstructure, capitate or clavate on distal faces with each caput or expanded apex and the rest of the surface with plicate microstructure (Figs. 4E &  4F), 27–30 µm.

**Additional specimen examined (paratype):–**BRAZIL. Espíritu Santo: Santa Teresa, Reserva Biológica Augusto Ruschi, trilha da cachoeira, 19°55′14″S, 40°33′37″W, 750–850 m, 2 Dec 2008, *Salino et al. 13978* (BHCB-n.v., P, fragment PMA).

**Habitat and distribution:–***S. agioneuma* grows in dense premontane wet forests at 750–860 m in Atlantic forest vegetation. It is known only from two collections made along forest trails in the Reserva Biológica Augusto Ruschi, Espíritu Santo.

**Etymology:–**The name derives from the Greek “*Agio pnévma*” meaning holy spirit and is in reference to the Brazilian State from which it was collected.

**Conservation status:–***S. agioneuma* was collected in a biological reserve, where it may be adequately conserved. Nevertheless, given that only two collections are known of this newly described taxon, it is considered Data Deficient (DD) according to IUCN categories and criteria (IUCN, 2012).

### Discussion

*S. agioneuma* is a very distinct species characterized by its decumbent to ascending habit, ventral and dorsal, stout rhizophores, upper surfaces of leaves usually with idioblasts, median leaf apices long-aristate with each arista denticulate and tipped by one to three teeth, and lemon yellow megaspores. In addition, the acroscopic halves of the lateral leaves are twice as wide as the basiscopic halves (i.e., 2.0 × 1.0 mm wide), while the inner halves of the median and axillary leaves are 1∕2 wider than the outer halves (i.e., 1.5 × 1.0 mm wide) and the halves of the dorsal and ventral sporophylls that overlap with each other are 1∕2 smaller than the halves that do not overlap.

*Selaginella agioneuma* is morphologically similar to *S*. *contigua* by having a decumbent to ascending habit, idioblasts on the upper leaf surfaces, median leaf apices which taper into a long arista (which are long-acuminate to short- or long-aristate in *S*. *contigua*), and yellow megaspores. In addition, it somewhat resembles *S*. *nanuzae* Valdespino, and *S. mendocae* Hieron., because of its habit, stem width (including lateral leaves), and overall leaf shape. Nevertheless, *S*. *agioneuma* differs from *S. contigua* by the characters listed under the diagnosis, while from *S*. *nanuzae* it is easily distinguished by its smooth, green upper and lower leaf surfaces (vs. upper surfaces bumpy and green, and lower surfaces corrugate to striate and silvery green), median leaf inner margins dentate to short-ciliate throughout and the outer margins entire along proximal 1∕2, otherwise dentate to short-ciliate along distal 1∕2 (vs. margins long-ciliate throughout and more obviously so on outer margins, where the cilia are half to one time longer than the inner cilia), the bases subcordate and glabrous (vs. truncate or oblique with outer bases auriculate, the auriculae tufted with 10–18 cilia), the axillary leaves 4.0–5.0 × 1.5–3.0 mm (vs. 1.5–2.6 × 0.8–1.1 mm); the lateral leaf bases denticulate along proximal 1∕2, then becoming entire and again denticulate along distal 1∕4 or denticulate throughout (vs. long-ciliate along proximal 3∕4, otherwise denticulate distally), basiscopic margins entire along proximal 3∕4, otherwise denticulate on distal 1∕4 (vs. long-ciliate along proximal 1∕8, otherwise entire distally or denticulate on distal 1∕8); apices acute to apiculate (vs. falcate and acute or gradually tapering and acute). *Selaginella agioneuma* differs from *S*. *mendocae* (which has obscure idioblasts on the upper and lower surfaces of the leaves and sporophylls, and yellow megaspores) by the upper surfaces of the leaf smooth (vs. strongly corrugate), median leaf outer margins entire along proximal 1∕2, otherwise dentate to short-ciliate along distal 1∕2 (vs. long-ciliate throughout), long-aristate (vs. cuspidate to shortly acuminate) apices, each arista 1∕4–1∕2 the length of the laminae (vs. 1∕20–1∕10), 0.5–1.5 (vs. 0.1 or 0.2) mm long, and oblong or broadly ovate-oblong (vs. oblong) lateral leaves with the basiscopic bases entire (vs. long-ciliate along ca. 1∕10 of the proximal base), and with broad and abruptly tapering apices ending in an acute tip (vs. truncate).

Additionally, similarities in habit and stem width (including lateral leaves) might cause *S. agioneuma* to be confused with other Brazilian species of the genus such as *S. breynii* Spring, *S. falcata* (P. Beauv.) Spring*,* and *S. flexuosa* Spring. Nevertheless, *S. falcata* has ovate-deltate median leaf with bases cordate, each lobe ciliate, margins ciliate, and acuminate toothed apices that tend to lift up, the acroscopic 1∕4 of the lateral leaf ciliate, and axillary leaves narrowly lanceolate to lanceolate. On the other hand, *S. breynii* has median leaf outer margins distinctly ciliate throughout, incurved midribs with the long-aristate apices interlocking with the distal portion of the leaf apices above, the outer bases ciliate, the lateral leaf proximal 1∕4 long-ciliate, otherwise distally dentate with the midribs on lower surfaces distinctly marked, the basiscopic margins long-ciliate along proximal 1∕8, the apices mostly gradually tapering into a broad acute tip, and the axillary leaf narrowly lanceolate to lanceolate. Finally, *S. flexuosa* has leaves upper surfaces distinctly corrugate, median leaf margins denticulate with long toothed aristae abruptly born at the apices, which frequently interlock with the laminae of the leaves above them, the lateral and axillary leaves margins entire to minutely denticulate, and the lower surfaces of the leaves with many, elongate, idioblasts.

**Table:** 

**Figure 5:**
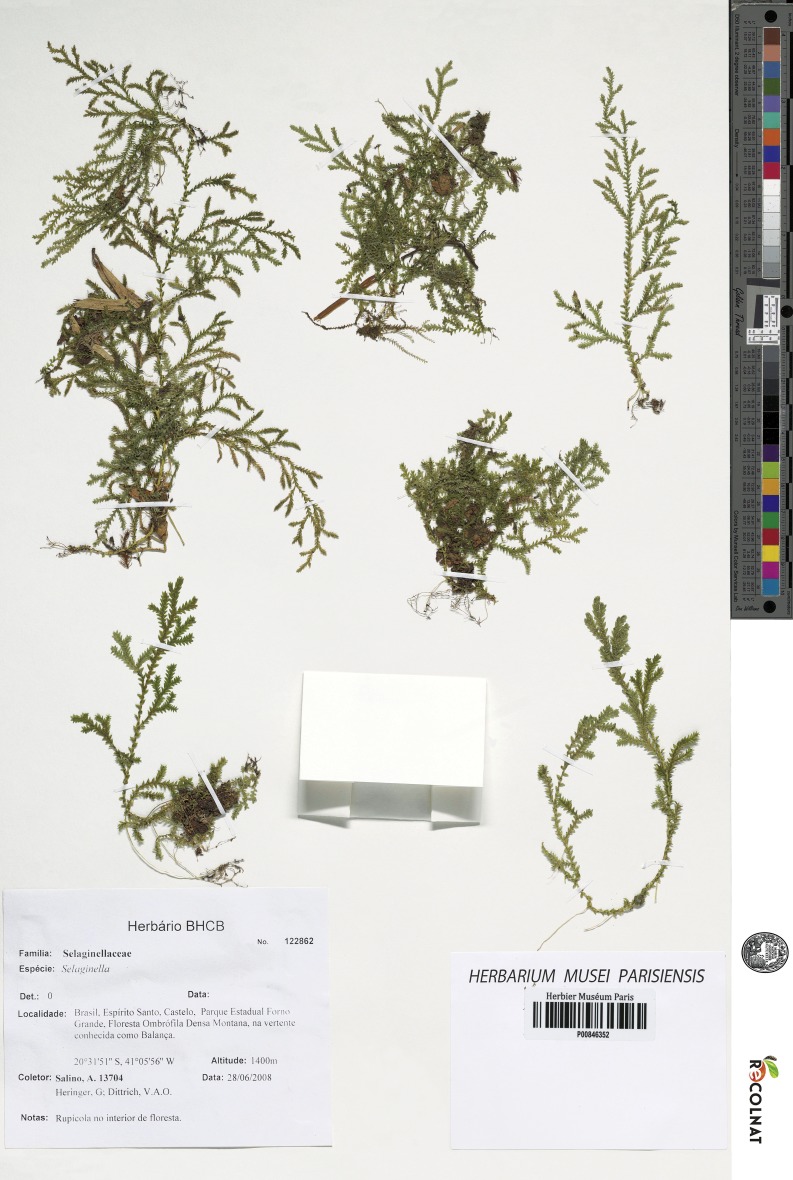
Scanned image of holotype of *S. magnafornensis*. *Salino et al. 13704* (P). Image courtesy of Muséum national d’Histoire naturelle, Paris, France.

**Figure 6:**
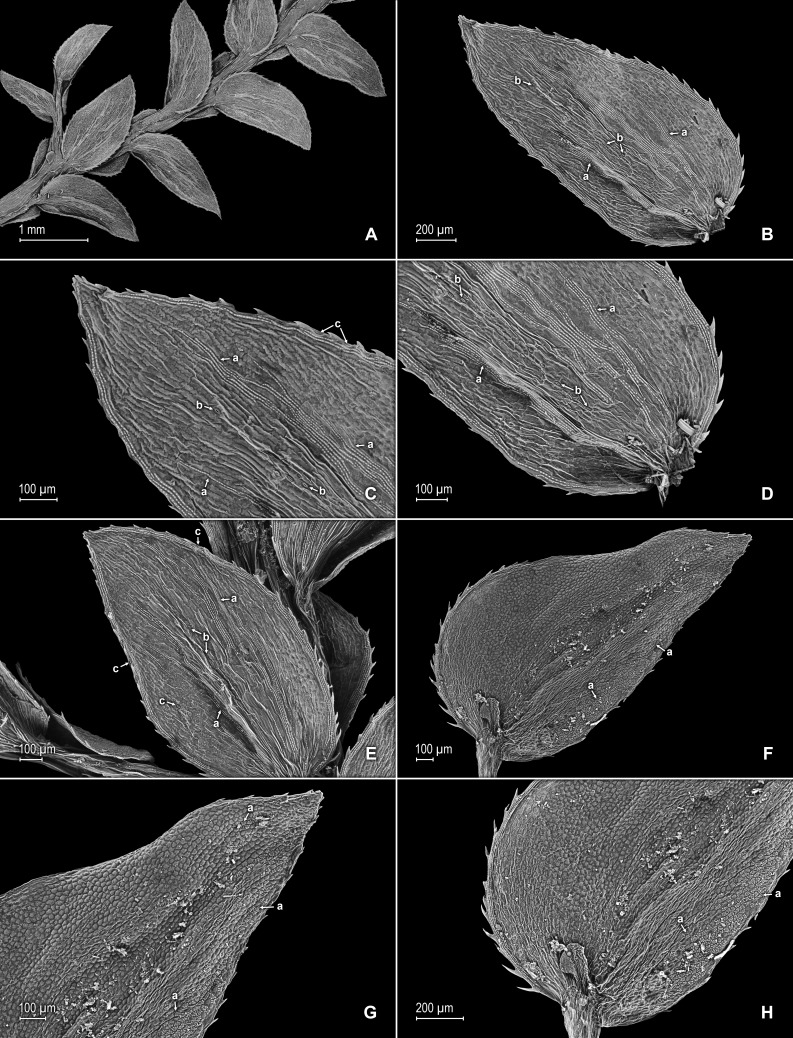
Scanning electron micrographs of branch section and leaves of *S. magnafornensis*. (A) Section of lower surface of stem branch showing lateral and axillary leaves. (B) Close-up of lateral leaf from stem branch, lower surface; note elongate and interconnected, papillate idioblast (a) on both side of midrib and stomata (b) along midrib. (C) Close-up distal portion of lateral leaf (same leaf shown in B); note elongate and interconnected, papillate idioblast (a) on both side of midrib and stomata along midrib (b) and margin (c). (D) Close-up of proximal portion of lateral leaf (same leaf shown in B); note elongate and interconnected, papillate idioblast (a) on both side of midrib and stomata along midrib (b). (E) Close up of axillary leaf from stem branch, lower surface; note elongate and interconnected, papillate idioblast (a) on both side of midrib and stomata along midrib (b) and margins (c). (F) Close-up of lateral leaf from stem branch, upper surface; note papillate cells (a). (G) Close-up of distal portion of lateral leaf (same leaf shown in F); note papillate cells (a). (H) Close-up of proximal portion of lateral leaf (same leaf shown in F); note papillate cells (a). (A–H) taken from holotype, *Salino et al. 13704* (P).

**Figure 7:**
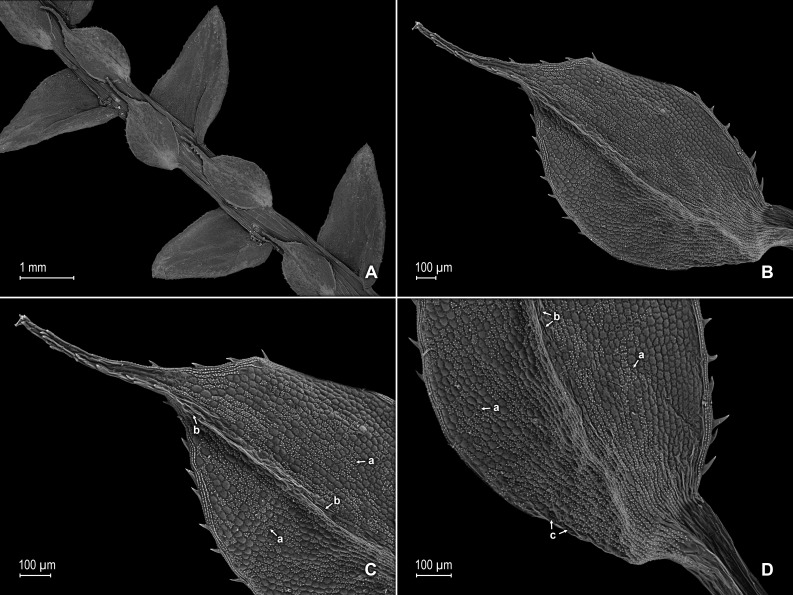
Scanning electron micrographs of branch section and leaves of *S. magnafornensis*. (A) Section of upper surface of stem branch showing lateral and median leaves. (B) Close-up of median leaf from stem branch, upper surface. (C) Close-up distal portion of lateral leaf (same leaf shown in B) showing aristate apex; note papillate cells (a) and stomata (b) along the midrib. (D) Close-up of proximal portion of lateral leaf (same leaf shown in B) showing base; note papillate cells (a) and stomata along midrib (b) and submarginal near base (c). (A–D) taken from holotype, *Salino et al. 13704* (P).

**Figure 8:**
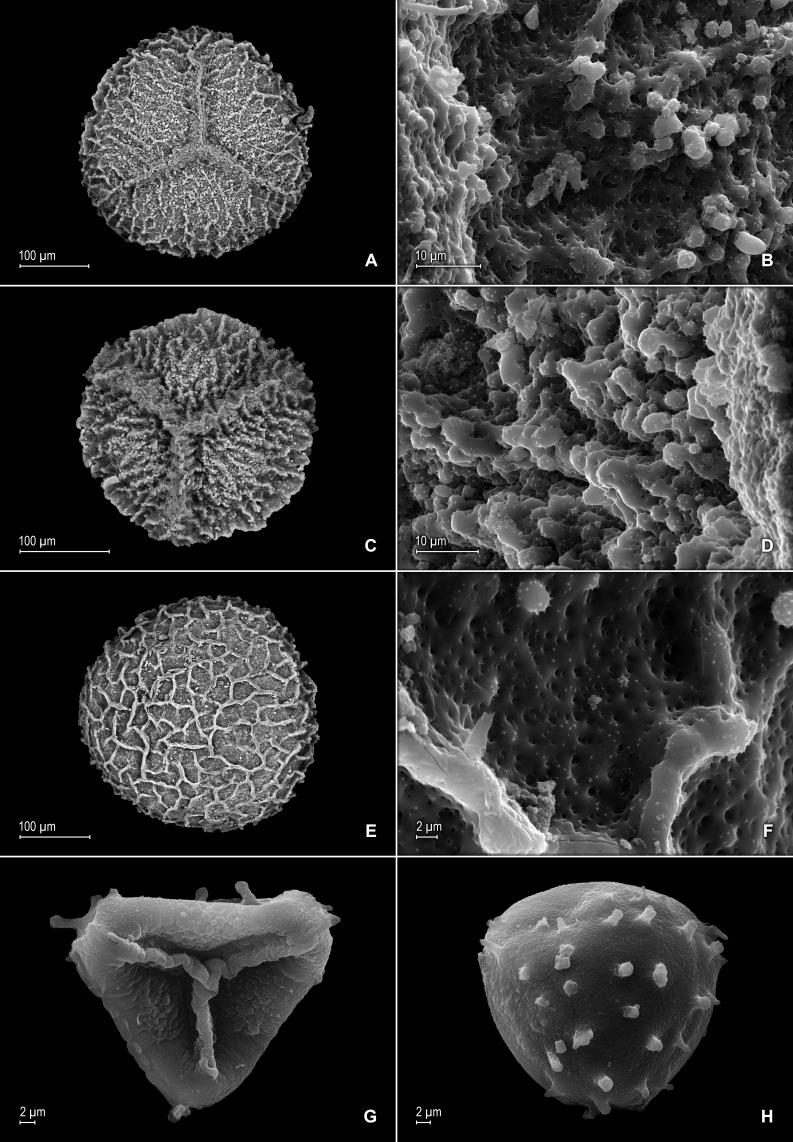
Scanning electron micrographs of mega- and microspores of *S. magnafornensis*. (A) Megaspore, proximal face. (B) Close-up of megaspore (same megaspore shown in A), proximal face. (C) Megaspore (less mature), proximal face. (D) Close-up of megaspore (same megaspore shown in C), proximal face. (E) Megaspore distal face. (F) Close-up of megaspore (same megaspore shown in E), distal face. (G) Microspore, proximal face. (H) Microspore, distal face. (A–H) taken from holotype, *Salino et al. 13704* (P).

***Selaginella magnafornensis*** Valdespino & C. López, sp. nov.
(Figs. 5–8)

**Type:–**Brazil. Espíritu Santo. Castelo, Parque Estadual Forno Grande, 20°31′51″S, 41°05′56″W, 1,400 m, 28 Jun 2008, *A. Salino, G. Heringer, & V.A.O. Dittrich 13704* (holotype: P!; isotypes: BHCB-n.v., fragment PMA!).

**LSID:**77178085-1.

**Diagnosis:–***S. magnafornensis* differs from slender forms of *S*. *flexuosa* by its smooth (vs. corrugate) upper leaf surface, ovate-oblong (vs. oblong) lateral leaves with acroscopic margins shortly ciliate to dentate along proximal 1∕3–1∕2 of each lamina (vs. denticulate along proximal 1∕3, otherwise entire), and ovate, ovate-oblong, ovate-cordate or narrowly ovate (vs. elliptic to oblong-elliptic) axillary leaves, and yellow (vs. white) megaspores.

**Description:–***Plants* terrestrial. *Stems* ascending, stramineous, 15–25 cm long, 0.4–0.8 mm diam., non-articulate, not flagelliform or stoloniferous, 2- or 3-branched. *Rhizophores* ventral, axillar, and dorsal, borne on proximal 1∕8–1∕3 of stems, stout, 0.1–0.5 mm diam. *Leaves* heteromorphic throughout, chartaceous to slightly coriaceous, strongly distant, upper surfaces smooth and green, lower surfaces smooth and silvery green. *Lateral leaves* perpendicular to the stems or slightly ascending, ovate to ovate-oblong, 1.7–2.5 × 0.8–1.5 mm; bases rounded to semicordate, glabrous, acroscopic bases strongly overlapping stems, basiscopic bases free from stems; acroscopic margins hyaline in a band one to six cells wide (usually one to three cells wide on basiscopic margins) with the cells elongate, straight-walled and papillate parallel to margins, papillae in one row over each cell lumen, shortly ciliate to dentate along proximal 1∕3–1∕2, otherwise denticulate apically, basiscopic margins narrowly hyaline to greenish hyaline in a band one to three cells wide, the cells as in acroscopic margins, denticulate; apices acute, variously tipped by two to four teeth; upper surfaces comprised of quadrangular or rounded, sinuate-walled cells, many of these along submarginal portion of basiscopic halves and apical 1∕4 of the laminae covered by 2–12 papillae, without idioblasts or stomata, lower surfaces comprised of elongate, sinuate-walled cells and many papillate idioblast on both sides of the midribs, the idioblasts three to six longer than surrounding cells and somewhat interconnecting as to appear veins (i.e., false veins), papillae in one row over each cell lumen, with stomata in one or two rows along proximal 3∕4 of the midribs and at least one or few scattered on proximal, submarginal 1∕2 of acroscopic margins and on distal 1∕3 of the margins. *Median leaves* ascending, ovate-elliptic, 1.0–1.8(2.0) × 0.8–1.4 mm; bases oblique, glabrous; margins hyaline in a band two to four cells wide, the cells elongate, straight-walled and papillate parallel to margins, papillae in one row over each cell lumen, margins dentate; apices long-aristate, each arista 1∕3–1∕2 the length of the laminae (0.4–0.7 mm long), dentate and variously tipped by one to four teeth; both surfaces without conspicuous idioblasts, upper surfaces comprised of quadrangular, rectangular or rounded, sinuate-walled cells, many of these covered by 6–14 papillae, with stomata in one row along distal 1∕2 of the midribs, sparsely present on submarginal portion along proximal 1∕4 of outer halves of the laminae, lower surfaces comprised of elongate, sinuate-walled cells, without stomata. *Axillary leaves* similar to lateral leaves or broadly ovate, 1.8–2.4 × 1.0–1.5 mm; bases rounded, glabrous; margins narrowly hyaline, margins dentate to denticulate; apices acute, variously tipped by one to three teeth; both surfaces as in lateral leaves, except for stomata along distal 1∕2 of the laminae and margins. *Strobili* terminal on main stem and branch tips, quadrangular, 3.0–8.0 cm. *Sporophylls* monomorphic, without a laminar flap, each with a well developed and seemingly glabrous keel (as observed with SM) along midribs, ovate-lanceolate, 0.9–1.2 × 0.5–0.7 mm; bases rounded; margins slightly hyaline, dentate; apices acuminate to aristate, each acumen or aristae 0.2–0.4 mm long, tipped by one or two teeth; *dorsal sporophylls* with upper surfaces green and cells as in median leaves, including idioblasts, lower surfaces silvery green and comprised of elongate, sinuate-walled cells; *ventral sporophylls* with both surfaces greenish-hyaline, comprised of elongate, sinuate-walled cells. *Megasporangia* in two ventral rows; *megaspores* yellow to lemon yellow, reticulate-rugulate and with a slightly developed equatorial flange on proximal faces with granulate to slightly echinulate, verrucate and perforate microstructure, reticulate on distal faces with close reticula and verrucate, slightly echinulate, and perforate microstructure (Figs. 8A–8F), 315–340 µm. *Microsporangia* in 2 dorsal rows; *microspores* orange, rugulate on proximal faces with slightly verrucate to echinulate and perforate microstructure, capitate or clavate on distal faces with each caput or expanded apex and the rest of the surface with plicate, rugulate, and perforate microstructure (Figs. 8G &  8H), 25–32 µm.

**Habitat and distribution:–***S. magnafornensis* grows as epipetric in dense montane wet forests at 1,400 m in Atlantic forest vegetation. It is known only from the type collection made in Parque Estadual Forno Grande, Espíritu Santo.

**Etymology:–**The specific epithet is a compound word derived from the Latin “*magna*”, meaning big (“grande” in Portuguese), and “*furnus*”, meaning oven (“forno” in Portuguese), and alludes to the name of the park where the species was collected.

**Conservation status:–***S. magnafornensis* is known from a single collection made in a National Park in Brazil, which presumably is well-protected. However, at this point the species is best treated as Data Deficient (DD) according to IUCN categories and criteria (IUCN, 2012).

### Discussion

*S. magnafornensis* is a very distinct species with slender stems with well-spaced branches (1.0–1.5 cm apart) that bear strobili at their tips. It does not seem to have close morphological affinities with other Brazilian *Selaginella* species but its creeping stems, somewhat stiff and long-aristate median leaves are reminiscent of slender forms of *S*. *flexuosa*, however, the characters listed in the diagnosis set them apart.

**Table:** 

**Figure 9:**
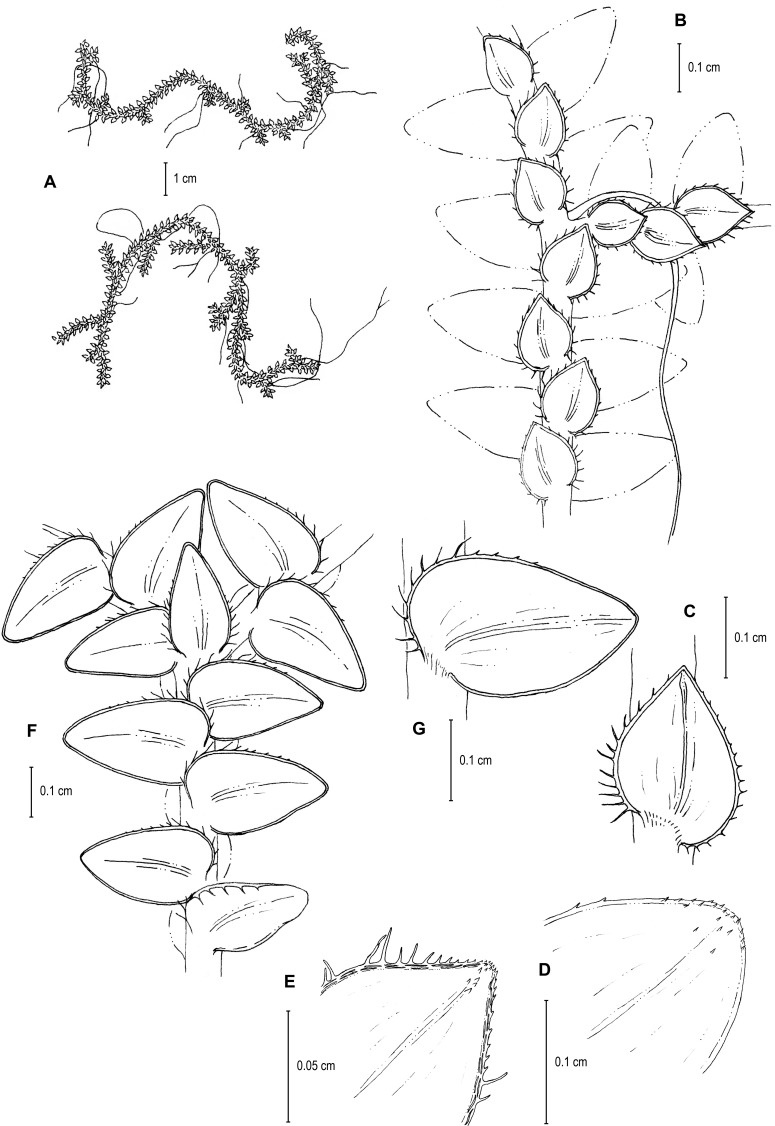
Line drawing of holotype of *S. ventricosa*. (A) Habit. (B) Upper surface of stem showing median and outline of lateral leaves. (C) Median leaf, upper surface. (D) Distal portion of lateral leaf upper surface showing teeth-like or prickle projections near the apex. (E) Distal portion of median leaf upper surface showing teeth-like or prickle projections near the apex. (F) Lower surface of stem showing lateral leaves and axillary leaf. (G) Lateral leaf, lower surface. (A–G) from *Terra-Araujo et al. 1320* (INPA). Illustration made by Rubén Lozano.

**Figure 10:**
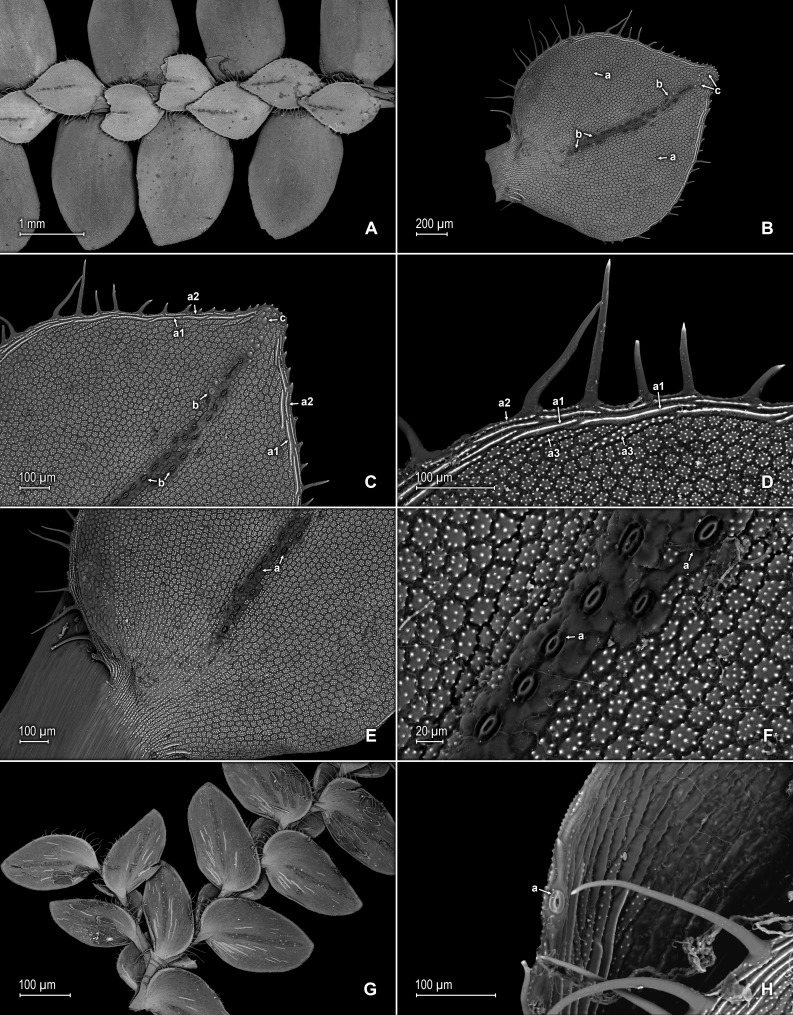
Scanning electron micrographs of branch sections and leaves of *S. ventricosa*. (A) Section of upper surface of stem showing median and lateral leaves. (B) Close-up of median leaf, upper surface; note, papillate cells (a), stomata (b) along midrib, and hairs (c) on distal portion of lamina, and ciliate inner margin. (C) Close-up of mid-distal portion of median leaf (same leaf shown in B); note, elongate, idioblast-like marginal cells with jointed and line-like (a1) or individual (a2) rows of papillae, stomata (b) along midrib, and hairs (c) on distal portion of lamina, and ciliate inner margin. (D) Acroscopic and distal portion of the inner margins of median leaf (same leaf shown in B) showing cilia; note, many papillae on each lumen of roundish to quadrangular cells, elongate, idioblast-like marginal cells with jointed and line-like (a1) or one (a2) or two rows of individual (a3) papillae. (E) Close up of proximal portion of median leaf (same leaf shown in B); note, cell lumina without or with many papillae and stomata (a) along midrib. (F) Close-up of median leaf (same leaf shown in B); note, cell lumina with many papillae and stomata (a) along midrib. (G) Section of lower surface of stem showing lateral and axillary leaves; note, elongate idioblasts. (H) Close-up of median leaf, lower surface; note stomata (a) on margin and elongate, sinuate-walled, and entire or papillate cells. (A–H) taken from holotype, *Terra-Araujo et al. 1320* (INPA).

**Figure 11:**
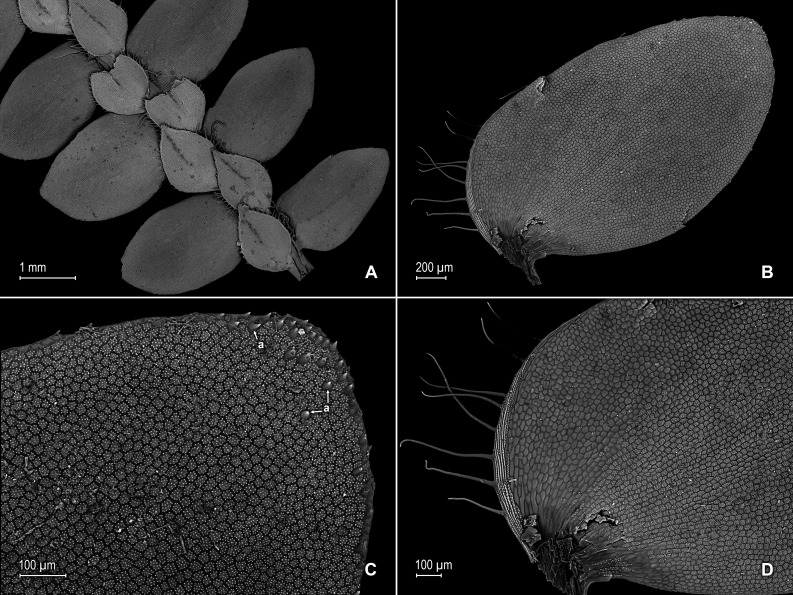
Scanning electron micrographs of branch section and leaves of *Selaginella ventricosa*. (A) Section of upper surface of stem showing lateral and median leaves. (B) Close-up of lateral leaf, upper surface. (C) Close-up of distal portion of lateral leaf (same leaf shown in B); note, many papillae on each cell lumen and hairs (a) on apical region. (D) Close-up of proximal portion of lateral leaf (same leaf shown in B); note, ciliate margin and roundish to quadrangular cells with or without many papillae on each cell lumen. (A–D) taken from holotype, *Terra-Araujo et al. 1320* (INPA).

**Figure 12:**
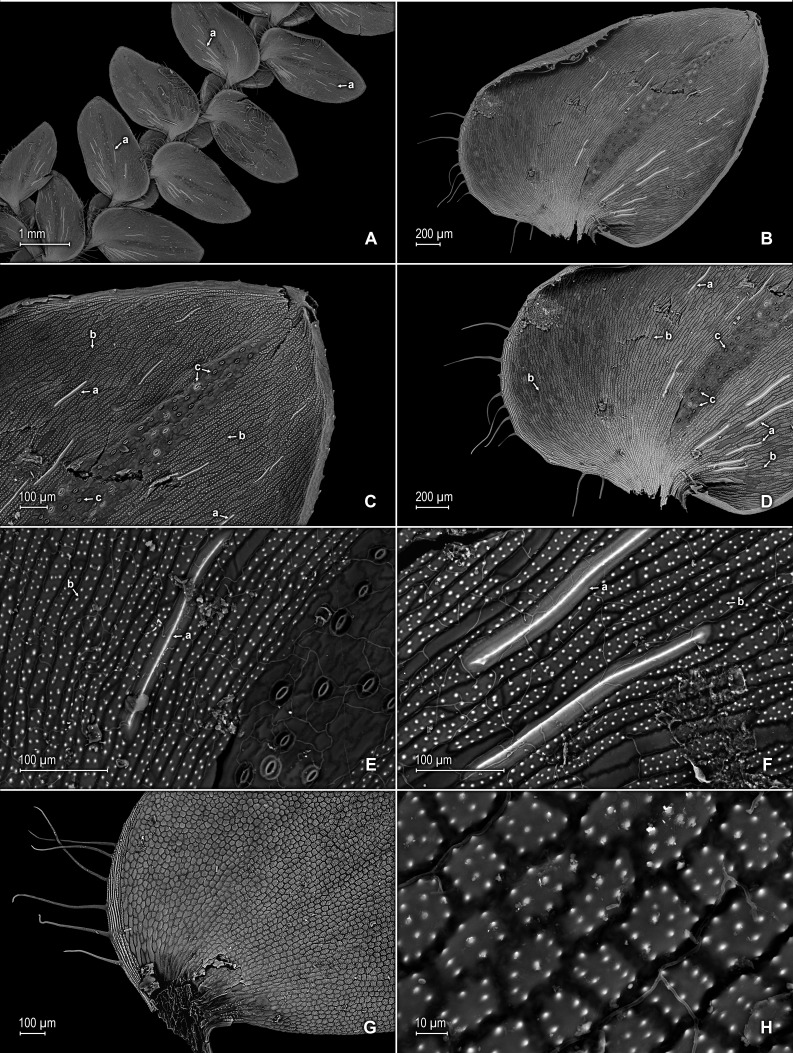
Scanning electron micrographs of branch section and leaves of *S. ventricosa*. (A) Section of lower surface of stem showing lateral leaves and portions of median leaves; note idioblasts (a). (B) Close-up of lateral leaf, lower surface. (C) Close-up distal portion of lateral leaf (same leaf shown in B); note, idioblasts with a single elongate papilla (a), elongate cells with many papillae mostly in two rows (b), and stomata (c) along midrib. (D) Close-up of proximal portion of lateral leaf (same leaf shown in B); note, idioblasts with a single elongate papilla (a), elongate cells with many papillae mostly in two rows (b), and stomata (c) along midrib. (E) Close-up of same view as in (D); note, idioblasts with a single elongate papilla (a), elongate cells with many papillae mostly in two rows (b), and stomata. (F) Close-up of same view as in (D); note, idioblasts with a single elongate papilla (a) and elongate cells with many papillae mostly in two rows (b). (G) Close-up of proximal portion of lateral leaf, upper surface; note cells lumen mostly with papillae. (H) Close-up portion of lateral leaf, upper surface; note cells lumen with papillae. (A–H) taken from holotype, *Terra-Araujo et al. 1320* (INPA).

***Selaginella ventricosa*** Valdespino & C. López, sp. nov.
(Figs. 9–12)

**Type:–**BRAZIL. Roraima: [Mpio.] Caracarai, Parque Nacional Serra da Mocidade, 01°71′84.3″N, 61°75′37″W, 1,732 m, 3 Feb 2016, *M.H. Terra-Araujo*, *R. Braga-Neto & A. Vicentini 1320* (holotype: INPA!; isotype: PMA!).

**LSID:**77178086-1.

**Diagnosis:–***S. ventricosa* differs from *S*. *microphylla* (Kunth) Spring by its leaves with extended (vs. usually with reflexed) margins, broadly acute (vs. acute) median leaves and apices tipped by four to six teeth (vs. by three to five long cilia), with (vs. without) stomata on the upper surfaces, and stomata lacking (vs. present) on the lower surfaces. It also differs from *S*. *microphylla* by the median leaf inner hyaline margins along distal 1∕2 and the outer hyaline margins along distal 2∕3 with the papillae of the cells interconnected (vs. papillae distinct) and appearing (vs. not) idioblast-like, and lower surfaces of lateral leaves with many (vs. some) papillate idioblasts, the idioblasts long (vs. short) with papillae mostly in 2 (vs. two to four) rows or in a single interconnected row (vs. always in two or more distinct rows) of papillae.

**Description:–***Plants* epiphytic. *Stems* creeping, stramineous, 7.0–14.5 cm long, 0.4–0.6 mm diam., non-articulate, not flagelliform or stoloniferous, one- to two-branched. *Rhizophores* axillary, borne throughout the stem, filiform, 0.1–0.3 mm diam. *Leaves* heteromorphic throughout, membranaceous, both surfaces glabrous, upper surfaces green, lower surfaces silvery green. *Lateral leaves* spreading, broadly ovate or ovate-oblong, 2.0–2.7 × 1.2–2.2 mm; bases rounded, glabrous, acroscopic bases strongly overlapping the stems, basiscopic bases free from stems; acroscopic margins hyaline along proximal 1∕3 in a band three to seven cells wide with the cells elongate, straight-walled and papillate parallel to margins, papillae in two rows over each cell lumen, long-ciliate along proximal 3∕4, otherwise denticulate distally; basiscopic margins greenish, comprised of quadrangular, sinuate-walled, glabrous and papillate cells, short-ciliate along proximal 1∕8; otherwise entire distally or denticulate on distal 1∕8, apices broadly acute to obtuse, with prickles or tooth-like projections on the upper surface; upper surfaces comprised of quadrangular or rounded, sinuate-walled cells, many of these covered by 7–20 papillae, without idioblasts or stomata, lower surfaces comprised of elongate, sinuate-walled cells, with many of these papillate and with idioblasts on both side of the midribs (more so on basiscopic halves of the laminae), papillae in two to three rows over each cell lumen, with stomata in three to four rows along midribs. *Median leaves* distant on the main stem, ascending, broadly ovate to ovate-orbicular, 0.8–1.4 × 0.7–1.3 mm; bases truncate to oblique, glabrous, inner margins hyaline in a band two to six cells wide, the cells elongate, straight-walled and papillate parallel to margins, papillae in one row over each cell lumen and often interconnected along distal 1∕2 and appearing as a single line or idioblast, long-ciliate along proximal 4∕5, otherwise dentate to serrate on distal 1∕5, outer margins greenish along proximal 1∕3 and the cells similar to those of the leaf upper surfaces, otherwise hyaline on distal 2∕3 in a band one or two cells wide with the cells similar to those found on distal 1∕2 of inner margins, entire along proximal 1∕3, except for a single long-ciliate at proximal most portion near the base, otherwise short-ciliate on median 1∕3 and dentate to serrate on distal most 1∕3; apices broadly acute, 0.1–0.2 mm long, variously tipped by four to six teeth and with tooth-like or prickle projections along its length on upper surfaces; upper surfaces comprised of quadrangular, rectangular or rounded, sinuate-walled cells, many of these covered by 5–15 papillae, with stomata in one to two rows along distal 1∕2–2∕3 of the midribs ending before distal most apical portion of the lamina and along proximal 1∕3 of outer margins, lower surfaces comprised of elongate, sinuate-walled cells, with papillate idioblasts concentrated on proximal 1∕3 of the outer margins, the papillae in two rows of each cell lumen, without stomata. *Axillary leaves* narrowly ovate or ovate-oblong, 1.3–1.6 × 1.0–1.3 mm; bases truncate, glabrous; margins hyaline, long-ciliate along proximal 2∕3, especially on inner margins, otherwise denticulate on distal 1∕3; apices acute to broadly acute, variously tipped by one to three teeth; both surfaces as in lateral leaves. *Strobili*, *sporophylls*, *sporangia* and *spores* not observed because plants where unfertile.

**Habitat and distribution:–***S. ventricosa* grows as an epiphyte in upper montane Amazon forests at 1,732 m in an isolated tepui complex in the southern portion of the Guiana Plateau; it is only known from the type locality in Roraima State, Brazil, where it might be a local endemic. Recently, other plant and insect species were described from the Serra da Mocidade mountain range (see for example Bastos, Sierra & Zartman, 2016; Da Silva-Neto, Aldrete & Rafael, 2016; Dantas & Hamada, 2017) that also has an herpetofauna richness comparable to other Guiana Shield mountains (Moraes et al., 2017), thus highlighting its biodiversity importance within the region.

**Etymology:–**The species name is derived from the Latin “*ventricosus*”*,* meaning swollen, especially on one side, and refers to the median leaf outer halves somewhat ventricose or bulging.

**Conservation status:–***S. ventricosa* was collected in Serra da Mocidade National Park, a poorly explored and isolated tepui complex of the Brazilian Guiana Shield, that is closed to the public and thus this species is considered as Data Deficient (DD), according to IUCN categories and criteria (IUCN, 2012).

### Discussion

*S. ventricosa* is characterized by its broadly ovate to ovate-orbicular median leaves with asymmetric halves, the outer halves 1∕4 or less wider than the inner halves and somewhat appearing ventricose or bulging, truncate to oblique bases. It is further characterized by its median leaf inner margins long-ciliate along proximal 4∕5, the inner margins distal 1∕2 and the outer margins distal 2∕3 hyaline, composed of papillate cells with the papillae interconnected appearing as a single line or idioblast, stomata along proximal 1∕3 of outer margins, broadly acute apices tipped by four to six teeth, and each apex upper surface with prickles or tooth-like projections along its length and with many of the cells lumina covered by 5–15 papillae.

*Selaginella ventricosa* is a unique species that might be confused with *S*. *microphylla*, a taxon within the species group of the same name that is also found in Brazil, by its similar creeping habit and leaves. *S. ventricosa* is set easily aside from the latter by the characters listed in the diagnosis and by its median leaves with truncate to oblique (vs. distinctly oblique) bases, with the inner margins long-ciliate along proximal 4∕5 and outer margins short-ciliate on median 1∕3 (vs. inner and outer margins usually long-ciliate throughout), and the upper surfaces with 5–15 [vs. 1–4(6)] papillae.

**Table:** 

**Figure 13:**
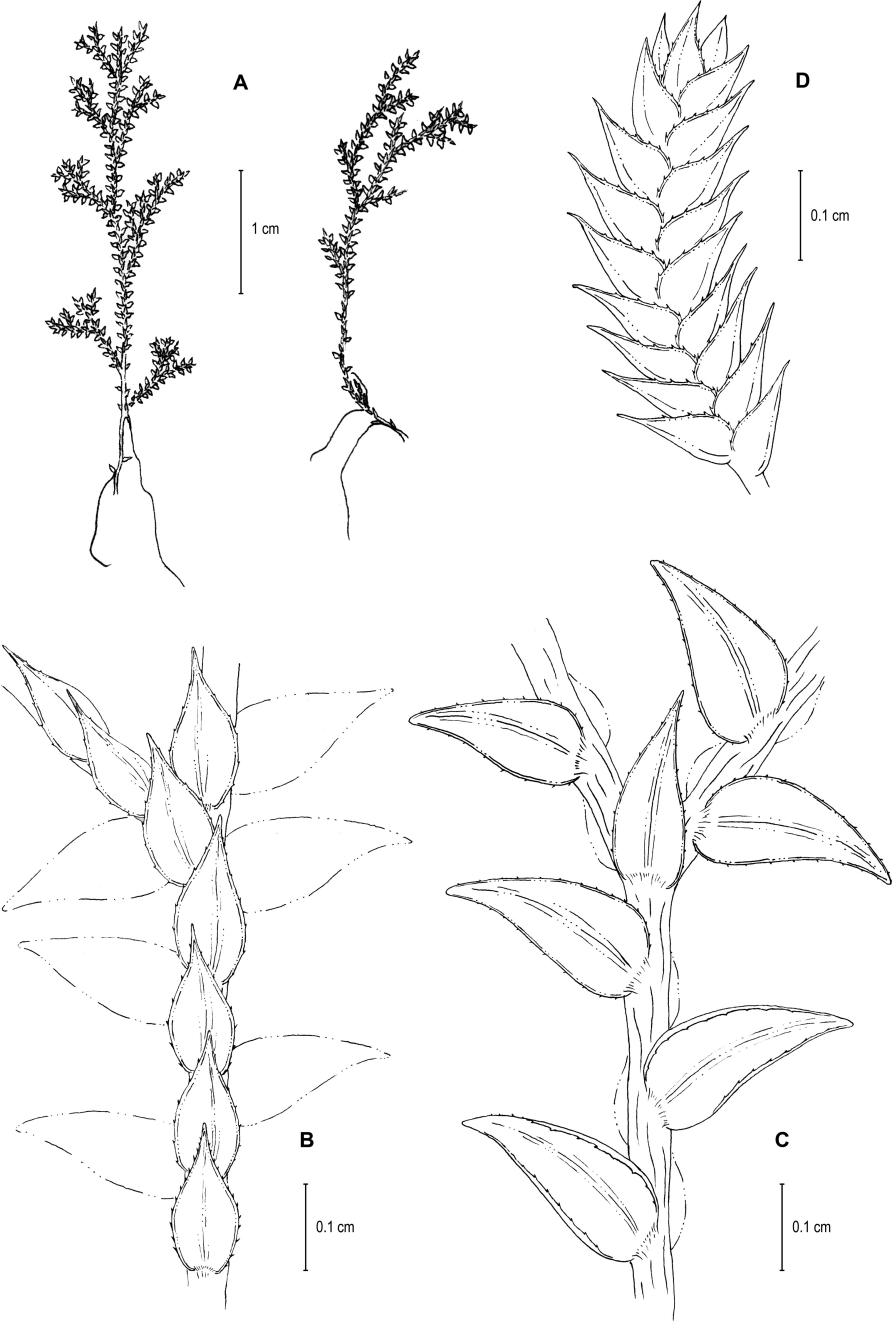
Line drawing of holotype of *S. zartmanii*. (A) Habit. (B) Upper surface of stem showing median leaves and outline of lateral leaves. (C) Lower surface of stem showing lateral leaves and axillary leaf. (D) Close-up of strobilus. (A–D) from *Terra-Araujo et al. 1320* (INPA). Illustration made by Rubén Lozano.

**Figure 14:**
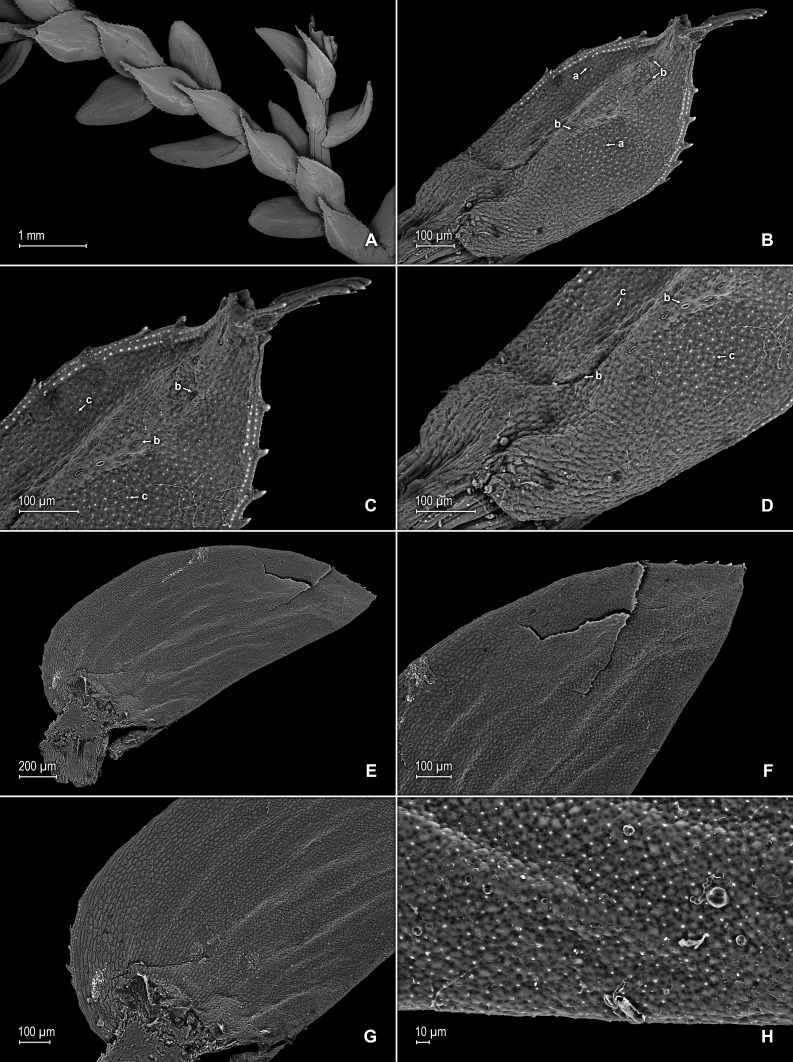
Scanning electron micrographs of branch section and leaves of *S. zartmanii*. (A) Section of upper surface of stem showing median and lateral leaves. (B) Close-up of median leaf, upper surface; note, papillate cells (a) and stomata (b) along midrib. (C) Close-up of mid-distal portion of median leaf (same leaf shown in B); note, elongate, idioblast-like marginal cells with a single row of papillae, stomata (b) along midrib, and some cells with a single papilla (c) on cell lumen. (D) Close-up of mid-proximal portion of median leaf (same leaf shown in B); note, elongate, idioblast-like marginal cells with a single row of papillae stomata (b) along midrib, and some cells with a single papilla (c) on cell lumen. (E) Close up lateral leaf, upper surface. (F) Close-up of distal portion of lateral leaf (same leaf shown in E); note, some cell lumen with a single papilla. (G) Close-up of proximal portion of lateral leaf (same leaf shown in E). (H) Close-up of portion of basiscopic half of lateral leaf (same leaf shown in E); note, some cell lumen with a single papilla. (A–H) taken from holotype,* Zartman 8586* (INPA).

**Figure 15:**
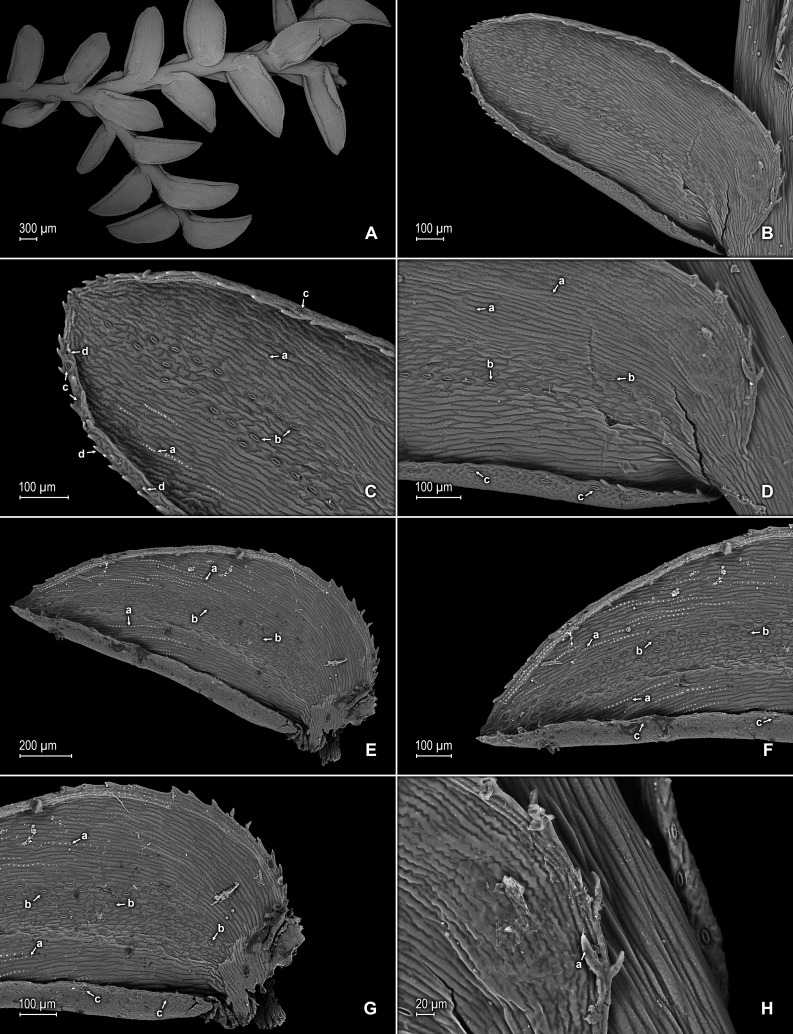
Scanning electron micrographs of branch section and leaves of *S. zartmanii*. (A) Section of lower surface of stem showing lateral leaves. (B) Close-up of lateral leaf, lower surface; note, revolute basiscopic margin. (C) Close-up of mid-distal portion of lateral leaf (same leaf shown in B); note, elongate and papillate, idioblast (a), stomata (b) along midrib and basiscopic margin revolute with marginal stomata (c) and tooth-like projections (d) on upper surface. (D) Close-up of mid-proximal portion of lateral leaf (same leaf shown in B); note, some elongate cells with papillae in a single row (a), stomata (b) along midrib, and marginal and submarginal stomata (c) on upper surface of basiscopic margin. (E) Close up lateral leaf, lower surface; note, elongate and papillate idioblasts (a) and stomata (b) along midrib. (F) Close-up of distal portion of lateral leaf (same leaf shown in E); note, elongate and papillate idioblasts (a), stomata (b) along midrib and marginal stomata (c) on upper surface of basiscopic margin. (G) Close-up of proximal portion of lateral leaf (same leaf shown in E); note, elongate and papillate idioblasts (a), stomata (b) along midrib and marginal stomata (c) on upper surface of basiscopic margin. (H) Close-up of portion of acroscopic base of lateral leaf; note, cilia (a). (A–H) taken from holotype,* Zartman 8586* (INPA).

**Figure 16:**
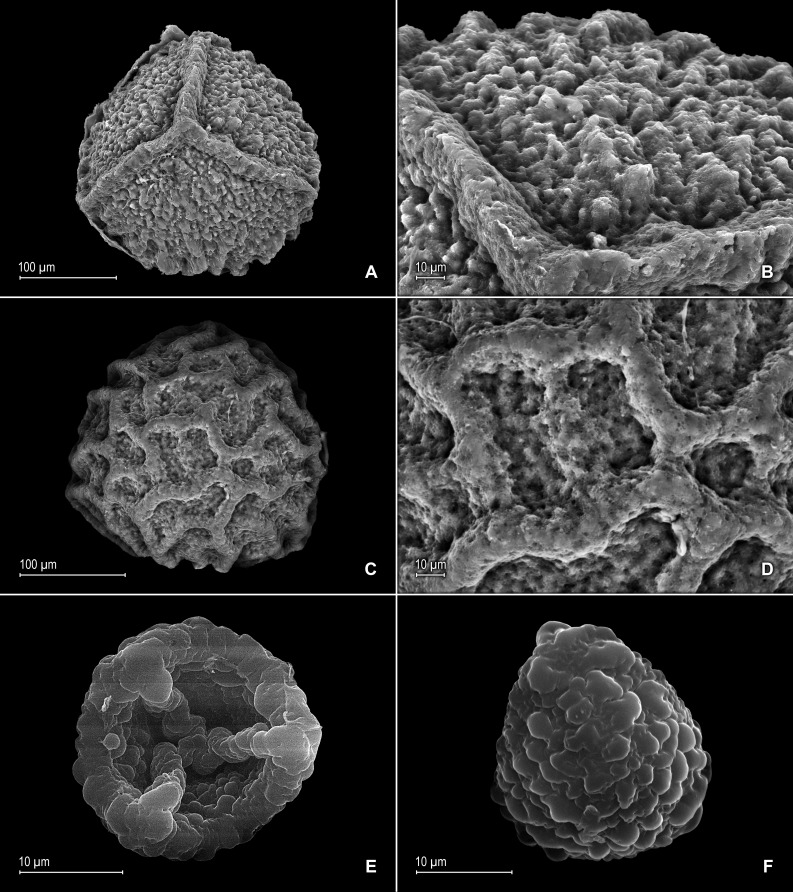
Scanning electron micrographs of mega- and microspores of *S. zartmanii*. (A) Megaspore, proximal face. (B) Close-up of megaspore, proximal face. (C) Megaspore, distal face. (D) Close-up of megaspore, distal face. (E) Microspore, proximal face. (F) Microspore, distal face. (A–F) taken from holotype,* Zartman 8586* (INPA).

***Selaginella zartmanii*** Valdespino, C. López & A.M. Sierra, sp. nov.
(Figs. 13–16)

**Type:–**BRAZIL. Amazonas: [Mpio.] São Gabriel da Cachoeira, Reserva Morro dos Seis Lagos, Lago do Dragão, 0°17′06″N, 66° 40′36″W, 400–450 m, 27 Aug 2011, *C.E. Zartman 8586* (holotype: INPA!; isotype: PMA!).

**LSID:**77178087-1.

**Diagnosis:–***S. zartmanii* is very similar to and easily confused with *S*. *boomii* Valdespino from which it is set aside by its glabrous (vs. papillate) upper median leaf surfaces or only with few papillate cells along the midribs (vs. papillae throughout the leaf lamina), lumen of papillate cells each with one papilla (vs. two to four papillae), margins narrowly (vs. broadly) hyaline and composed of one or two (vs. two to four) elongate and papillate cells, short (vs. long) aristate apices which extend 1∕4 (vs. 1∕3 or more) the length of the lamina, lateral leaves basiscopic margins with (vs. without conspicuous) submarginal stomata on upper surfaces, the acroscopic margins on upper surfaces composed of quadrangular to rectangular and glabrous (vs. elongate and papillate) green (vs. hyaline) cells, each assembled in a band of one or two (vs. two to seven) cells, and with (vs. without) conspicuous submarginal stomata along distal 1∕3.

**Description:–***Plants* terrestrial or epipetric. *Stems* suberect to erect, stramineous or reddish, four to six cm long, 0.2–0.4 mm diam., non-articulate, not flagelliform or stoloniferous, 2 or 3-branched. *Rhizophores* axillary or ventro-axillary, borne on proximal 1∕3 of stems, filiform, 0.1–0.3 mm diam. *Leaves* heteromorphic throughout, membranaceous, both surfaces smooth, upper surfaces reddish, greenish or yellowish, lower surfaces reddish, silvery green or yellowish. *Lateral leaves* ascending, lanceolate-oblong or ovate-oblong, 1.3–1.8 × 0.4–0.9 mm; bases rounded, glabrous, acroscopic bases overlapping stems, basiscopic bases free from stems; acroscopic margins slightly hyaline in a band two or three cells wide with the cells elongate, straight-walled and papillate parallel to margins, papillae in one row over each cell lumen, serrate throughout, basiscopic margins greenish, often revolute, comprised of quadrangular, sinuate-walled cells, serrate throughout; apices acute, variously tipped by three to five teeth; both surfaces glabrous or upper surface sometimes with marginal prickles or tooth-like projections along the distal region of basiscopic margins, upper surfaces comprised of quadrangular or rounded, sinuate-walled cells (often difficult to distinguish because of waxy deposits), many of these covered by one or two papillae, without idioblasts or stomata or sometimes with submarginal to marginal stomata along both margins, lower surfaces comprised of elongate, sinuate-walled cells, with many of these papillate and idioblast-like on both sides of the midribs, papillae in one row over each cell lumen, with stomata in four or five rows along midribs. *Median leaves* ascending, distant to slightly imbricate in the apical region of stems and branches, ovate-elliptic or ovate, 0.6–1.2 × 0.4–0.7 mm; bases truncate and glabrous; margins slightly hyaline in a band one to three cells wide, the cells elongate, straight-walled and papillate parallel to margins, papillae in one row over each cell lumen, serrate throughout; apices aristate, arista 0.2–0.5 mm long, tipped by five or six teeth and with prickles or tooth-like projections along its length on upper surfaces; both surfaces without conspicuous idioblasts, upper surfaces comprised of quadrangular, rectangular or rounded, sinuate-walled cells (often difficult to distinguish because of waxy deposits), many of these covered by one or two papillae, with stomata in one or two rows along distal 1∕2 of the midribs, sparsely present on submarginal portion along proximal 1∕3 of outer halves of the laminae, lower surfaces comprised of elongate, sinuate-walled cells, without stomata. *Axillary leaves* lanceolate, 0.8–1.8 × 0.4–1.7 mm; bases rounded to subcordate, glabrous; margins serrate throughout; apices acute, variously tipped by one to three teeth; both surfaces as in lateral leaves. *Strobili* terminal on branch tips, one per branch, quadrangular, 0.9–5.0 cm long. *Sporophylls* monomorphic, without a laminar flap, each with a strongly developed and seemingly glabrous keel (as observed with SM) along midribs, ovate, 0.6–0.8 × 0.5–0.7 mm; bases truncate; margins slightly hyaline, dentate; apices acuminate to cuspidate, each acumen (cusp) 0.2–0.3 mm long, tipped by one to three teeth; *dorsal sporophylls* with upper surfaces green and cells as in median leaves, lower surfaces silvery green and comprised of elongate, sinuate-walled cells; *ventral sporophylls* with both surfaces hyaline or greenish hyaline, comprised of elongate, sinuate-walled cells. *Megasporangia* in two ventral rows; *megaspores* yellow, rugulate to coarsely rugulate-reticulate on proximal faces with a developed equatorial flange and spheroid or spheroid-perforate to spheroid-foveolate microstructure, coarsely reticulate or rugulate on distal faces with spheroid to spheroid-perforate or rugulate microstructure (Figs. 16A–16D), 200–265 µm. *Microsporangia* in two dorsal rows; *microspores* orange, gemmate on proximal faces with psilate microstructure, rugulate-gemmate or gemmate on distal faces with psilate and slightly foveolate microstructure (Figs. 16E & 16F), 22–25 µm.

**Additional specimens examined (paratypes):–**BRAZIL. Amazonas: Río Negro, [Mpio. São Gabriel da Cachoeira,] between Manaus and São Gabriel, Serra Curicuriari, 00°20″S, 66°50″W, 10 Jul 1979, *Poole 1948* (mixed coll., *1948b* at NY), Morro dos Seis Lagos, ca. 80 km N of São Gabriel, 00°20″N, 66°45″W, ca. 100 m, 18 Jul 1979, *Poole 2068* (NY-2 sheets), São Gabriel da Cachoeira, Mt. Morro dos Seis Lagos, Lago No. 2, 00°12″N, 66°00″W, 15 Oct 1987, *Stevenson et al. 702* (NY, PMA).

**Habitat and distribution:–***S. zartmanii* grows in lowland and mid elevations in tropical wet forests or dense premontane wet forests at 100–450 m in the upper Rio Negro at the Amazon basin. The type collection was made on rocks bordering an isolated lake formed by ancient volcanic plugs in the Morro dos Seis Lagos Biological Reserve, occurring in relative abundance on a geologically unique substrate for the region of an iron-rich reddish lateritic crust formation draining into the Igarapé–Mirim, a tributary of the Cauaburi River of the Rio Negro. This species has only been collected from in or around Mpio. São Gabriel da Cachoeira (including another isolated range in the region, the Serra Curicuriari), State of Amazonas, Brazil, which suggests *S*. *zartmanii* may be a local endemic to isolated outcrops of the upper Rio Negro. Further explorations of the Serras do Padre and Neblina complexes might reveal its presence there as well.

**Eponymy:–**This species honors Professor Dr. Charles Eugene Zartman (1970–), an outstanding American Bryologist who has made significant contributions in taxonomy and ecology of Neotropical mosses and liverworts, as well as in the ecology and botany of the Brazilian Amazon.

**Conservation status:–**This species grows in zones within the intersection of 3 protected areas: Pico da Neblina National Park, Morro dos Seis Lagos Biological Reserve, and Balaio Indigenous Park (Saraiva & Forzza, 2012) and was reported in the type collection information as consisting of abundant populations there. Therefore, it presently should be classified as a least concern (LC) taxon according to IUCN criteria (IUCN, 2012).

### Discussion

*Selaginella zartmanii* is easily confused with *S*. *boomii* because of their overall similar habit and leaves, and often having their lateral leaves basiscopic margins revolute when dry. In fact, Valdespino (2015b), included specimens here cited as paratypes of *S*. *zartmanii* under the latter. Nevertheless, a recent study conducted to identify the type collection of *S*. *zartmanii* prompted us to reassess the circumscription of *S*. *boomii* and to segregate from it those specimens from São Gabriel da Cachoeira, Amazonas State, Brazil, ascribing them to the former species. *Selaginella zartmanii* differs most noticeably from *S*. *boomii* by the characters listed in the diagnosis. In addition, as already advanced by Valdespino (2015b) specimens here assigned to *S*. *zartmanii* differ from typical *S*. *boomii* in having its megaspores proximal faces coarsely rugulate-reticulate (vs. slightly rugulate-reticulate to rugulate-granulate) with (vs. without) a slightly developed equatorial flange and spheroid to spheroid-perforate (vs. echinulate and perforate) microstructure and coarsely reticulate (vs. slightly rugulate-reticulate to rugulate-granulate) distal faces, the reticula with broad and coarse (vs. fainted or vestigial) muri and spheroid to spheroid-perforate (vs. echinulate and perforate) microstructure [see Figs. 16A–16D depicted here and Fig. 2A–D in Valdespino (2015b), for megaspore sculpturing pattern comparison]. This new species further differs from *S*. *boomii* by having the lower surfaces of the lateral leaves with scattered (vs. variously assembled in continuous rows of three to nine) idioblasts mostly along distal 1∕2 or occasionally few proximal (vs. throughout both sides) of each leaf midrib, and glabrous leaf base cells (vs. papillate and idioblasts-like with papillae in one or two rows on each cell lumen).

## Additional Information and Declarations

### Data Availability

The following information was supplied regarding data availability: Specimens cited are housed at the following herbaria: INPA, NY, P, PMA (acronyms according to Thiers, 2018).

### New Species Registration

The following information was supplied regarding the registration of a newly described species: *Selaginella agioneuma* Valdespino & C. López: 77178084-1; Selaginella magnafornensis Valdespino & C. López: 77178085-1; Selaginella ventricosa Valdespino & C. López: 77178086-1; Selaginella zartmanii Valdespino, C. López & A.M. Sierra: 77178087-1.
